# Supplementary material for: Cellulose Nitrates-Blended Composites from Bacterial and Plant-Based Celluloses
Source: Polymers (Basel). 2024 Apr 23;16(9):1183. doi: 10.3390/polym16091183 (PMC11085800; doi:10.3390/polym16091183)
Supplement: Supplementary file 1 [file polymers-16-01183-s001.zip › polymers-2968798-supplementary.pdf]

Supplementary Materials

Cellulose nitrates-blended composites from bacterial and plant-based celluloses

Yulia A. Gismatulina*, Vera V. Budaeva

Bioconversion Laboratory, Institute for Problems of Chemical and Energetic Technologies, Siberian Branch of the Russian Academy of Sciences (IPCET SB RAS), Biysk 659322, Russia

*Correspondence: julja.gismatulina@rambler.ru (Y.A.G.)

Table of Contents

Materials and Methods. Quality Attributes of Cellulose Samples **S2**

Materials and Methods. Nitration of Cellulose Samples **S2**

Materials and Methods. Nitration of Cellulose Samples with Mixed Sulfuric–Nitric Acids (MA) and Stabilization **S3**

Materials and Methods. Nitration of Cellulose Samples with Concentrated Nitric Acid in Methylene Chloride (NA+MC) and Stabilization **S3**

Materials and Methods. Calculation of CN Yield **S3**

Materials and Methods. Analysis of CN Samples **S4**

Figure S1. Beakers turned upside-down during the viscosity measurement of CN NA+MC (from the left to the right): CN BC, CN BC/OHC=70/30, CN BC/OHC=50/50, CN BC/OHC=30/70, and CN OHC **S4**

Materials and Methods

Quality Attributes of Cellulose Samples

The cellulose quality indicators were determined by standard analytical methods. The α-cellulose content was quantified as per the TAPPI standard by treating a weighed portion of cellulose with a 17.5 % NaOH solution and quantifying the undissolved residue after being washed with a 9.5 % NaOH solution and water, followed by drying. The content of acid-insoluble lignin was measured according to the TAPPI standard using 72 wt.% sulfuric acid. Pentosans were transformed in boiling 13 wt.% HCl solution into furfural, which was collected in the distillate and determined on a xylose-calibrated UNICO UV-2804 spectrophotometer (United Products and Instruments, Dayton, NJ, USA) at a wavelength of 630 nm using the orcinol-ferric chloride reagent. The ash content was determined by the TAPPI standard by incinerating a weighed portion of cellulose in a muffle furnace at 600 °C. The degree of polymerization (DP) was determined by the outflow time of cellulose solution in cadoxene (cadmium oxide in ethylenediamine) from a VPZh-3 viscometer (Ecroskhim Ltd., Saint-Petersburg, Russia) with a capillary diameter of 0.92 mm. The moisture was measured on an Ohaus MB23 moisture analyzer (OHAUS Corporation, Parsippany, NJ, USA).

All experiments were done in triplicate and data expressed as averages.

Nitration of Cellulose Samples

The cellulose samples were nitrated in 500-mL porcelain beakers under continuous stirring using an HS-50A-Set vertical stirring device (Daihan Witeg, Seoul, South Korea).

Nitration of Cellulose Samples with Mixed Sulfuric–Nitric Acids (MA) and Stabilization

A weighed portion of cellulose (7 g) was treated with industrial MA containing 14 % water. The mass ratio of substrate to mixed acid ranged from 1:40 for OHC to 1:160 for BC and 1:150 for blended cellulose samples; the process temperature was 25–30 °C and the process time was 40 min. Upon completion of the nitration, the cellulose nitrates (CNs) obtained by MA (CN MA) were washed with water and subjected to high-temperature stabilization as follows: treatment with water for 1 h at 85–95 °C; treatment with a 0.03 % sodium carbonate solution for 3 h at 85–95 °C; and treatment with water for 1 h at 85–95 °C.

Nitration of Cellulose Samples with Concentrated Nitric Acid in Methylene Chloride (NA+MC) and Stabilization

A weighed portion of cellulose (7 g) was treated with freshly distilled, concentrated nitric acid (99%) in the presence of methylene chloride (NA+MC) in a ratio of 20:80. Prior to the nitration, the cellulose sample was pre-wetted with half of the methylene chloride mass, afterwards the wetted sample was submerged into the working acid mixture prepared from the remainder of methylene chloride and nitric acid. The mass ratio of substrate to NA+MC ranged from 1:90 for OHC to 1:90 for BC and 1:80 for blended celluloses, with the process temperature being 25–30 °C and the process time being 30 min. Upon completion, the CN NA+MC samples were successively washed with methylene chloride and ethanol, followed by treatment with water for 1 h at 85-95 °C.

Calculation of CN Yield

The yield of CN (%) was calculated by Eq. (S1):

$$W = (m \times 100) / m_{initial} \tag{S1}$$

where W is the yield of CN (%), m is the CN sample weight (g), and $m_{initial}$ is the weight of the initial cellulose sample (g).

Analysis of CN Samples

Prior to analysis, the CN samples were dried for 1 h at 100 °C. The nitrogen content was measured by the ferrous sulfate method by which the CNs were saponificated with concentrated sulfuric acid, and the formed nitric acid is reduced with iron (II) sulfate to nitrogen oxide that generates, in excess of iron (II) sulfate, a [Fe(NO)]SO4 complex compound that colors the solution yellow-pink. The solubility of the CN samples in acetone (1 g CN in 50 mL acetone) was measured by filtering the acetone-insoluble residue of CN, followed by drying and weighing.

The viscosity of CNs was determined by measuring the flow time of a 2 % CN solution in acetone out of a VPZh-1 capillary column (OOO Ecohim, Saint-Petersburg, Russia). The solubility of CNs in an alcohol–ester mixture (0.5 g CN in 150 mL mixture) was measured by filtering the CN residue insoluble in the alcohol–ester mixture, followed by drying and weighing.

All the experiments were done in triplicate and the data expressed as averages.

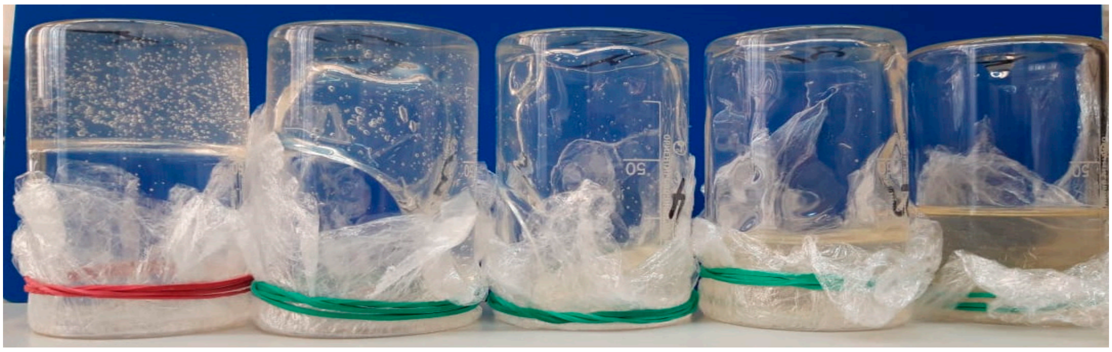

Figure S1. Beakers turned upside-down during the viscosity measurement of CN NA+MC (from the left to the right): CN BC, CN BC/OHC=70/30, CN BC/OHC=50/50, CN BC/OHC=30/70, and CN OHC
